# Supplementary material for: Resolving the full spectrum of human genome variation using Linked-Reads
Source: Genome Res. 2019 Apr;29(4):635–45. doi: 10.1101/gr.234443.118 (PMC6442396; doi:10.1101/gr.234443.118)
Supplement: Supplemental Material [file supp_gr.234443.118_Supplemental_longranger-2.2.zip › longranger-2.2.tar.bz2!/lr2.2-public/lib/stan/cmdstan/stan/doc/api/html/structstan_1_1gm_1_1statement__visgen.html]

Stan: stan::gm::statement\_visgen Struct Reference


|  |  |
| --- | --- |
|  | Stan  2.5.0  probability, sampling & optimization |


- Main Page
- Related Pages
- Namespaces
- Classes
- Files

- Class List
- Class Index
- Class Hierarchy
- Class Members

All Classes Namespaces Files Functions Variables Typedefs Enumerations Enumerator Friends Macros Pages

- stan
- gm
- statement\_visgen

Public Member Functions |
Public Attributes |
List of all members

stan::gm::statement\_visgen Struct Reference

`#include <generator.hpp>`

Inheritance diagram for stan::gm::statement\_visgen:

|  |  |
| --- | --- |
| Public Member Functions | |
|  | statement\_visgen (size\_t indent, bool include\_sampling, bool is\_var, bool is\_fun\_return, std::ostream &o) |
|  | |
| void | operator() (nil const &) const |
|  | |
| void | operator() (assignment const &x) const |
|  | |
| void | operator() (expression const &x) const |
|  | |
| void | operator() (sample const &x) const |
|  | |
| void | operator() (const increment\_log\_prob\_statement &x) const |
|  | |
| void | operator() (const statements &x) const |
|  | |
| void | operator() (const print\_statement &ps) const |
|  | |
| void | operator() (const reject\_statement &ps) const |
|  | |
| void | operator() (const return\_statement &rs) const |
|  | |
| void | operator() (const for\_statement &x) const |
|  | |
| void | operator() (const while\_statement &x) const |
|  | |
| void | operator() (const conditional\_statement &x) const |
|  | |
| void | operator() (const no\_op\_statement &) const |
|  | |
| Public Member Functions inherited from stan::gm::visgen | |
|  | visgen (std::ostream &o) |
|  | |

|  |  |
| --- | --- |
| Public Attributes | |
| size\_t | indent\_ |
|  | |
| bool | include\_sampling\_ |
|  | |
| bool | is\_var\_ |
|  | |
| bool | is\_fun\_return\_ |
|  | |
| Public Attributes inherited from stan::gm::visgen | |
| std::ostream & | o\_ |
|  | |

|  |  |
| --- | --- |
| Additional Inherited Members | |
| Public Types inherited from stan::gm::visgen | |
| typedef void | result\_type |
|  | |

## Detailed Description

Definition at line 1518 of file generator.hpp.

## Constructor & Destructor Documentation

|  |  |  |  |
| --- | --- | --- | --- |
| stan::gm::statement\_visgen::statement\_visgen | ( | size\_t | *indent*, |
|  |  | bool | *include\_sampling*, |
|  |  | bool | *is\_var*, |
|  |  | bool | *is\_fun\_return*, |
|  |  | std::ostream & | *o* |
|  | ) |  |  |

inline

Definition at line 1523 of file generator.hpp.

## Member Function Documentation

|  |  |  |  |  |  |
| --- | --- | --- | --- | --- | --- |
| void stan::gm::statement\_visgen::operator() | ( | nil const & |  | ) | const |

inline

Definition at line 1534 of file generator.hpp.

|  |  |  |  |  |  |
| --- | --- | --- | --- | --- | --- |
| void stan::gm::statement\_visgen::operator() | ( | assignment const & | *x* | ) | const |

inline

Definition at line 1536 of file generator.hpp.

|  |  |  |  |  |  |
| --- | --- | --- | --- | --- | --- |
| void stan::gm::statement\_visgen::operator() | ( | expression const & | *x* | ) | const |

inline

Definition at line 1548 of file generator.hpp.

|  |  |  |  |  |  |
| --- | --- | --- | --- | --- | --- |
| void stan::gm::statement\_visgen::operator() | ( | sample const & | *x* | ) | const |

inline

Definition at line 1553 of file generator.hpp.

|  |  |  |  |  |  |
| --- | --- | --- | --- | --- | --- |
| void stan::gm::statement\_visgen::operator() | ( | const increment\_log\_prob\_statement & | *x* | ) | const |

inline

Definition at line 1632 of file generator.hpp.

|  |  |  |  |  |  |
| --- | --- | --- | --- | --- | --- |
| void stan::gm::statement\_visgen::operator() | ( | const statements & | *x* | ) | const |

inline

Definition at line 1638 of file generator.hpp.

|  |  |  |  |  |  |
| --- | --- | --- | --- | --- | --- |
| void stan::gm::statement\_visgen::operator() | ( | const print\_statement & | *ps* | ) | const |

inline

Definition at line 1659 of file generator.hpp.

|  |  |  |  |  |  |
| --- | --- | --- | --- | --- | --- |
| void stan::gm::statement\_visgen::operator() | ( | const reject\_statement & | *ps* | ) | const |

inline

Definition at line 1673 of file generator.hpp.

|  |  |  |  |  |  |
| --- | --- | --- | --- | --- | --- |
| void stan::gm::statement\_visgen::operator() | ( | const return\_statement & | *rs* | ) | const |

inline

Definition at line 1685 of file generator.hpp.

|  |  |  |  |  |  |
| --- | --- | --- | --- | --- | --- |
| void stan::gm::statement\_visgen::operator() | ( | const for\_statement & | *x* | ) | const |

inline

Definition at line 1696 of file generator.hpp.

|  |  |  |  |  |  |
| --- | --- | --- | --- | --- | --- |
| void stan::gm::statement\_visgen::operator() | ( | const while\_statement & | *x* | ) | const |

inline

Definition at line 1708 of file generator.hpp.

|  |  |  |  |  |  |
| --- | --- | --- | --- | --- | --- |
| void stan::gm::statement\_visgen::operator() | ( | const conditional\_statement & | *x* | ) | const |

inline

Definition at line 1718 of file generator.hpp.

|  |  |  |  |  |  |
| --- | --- | --- | --- | --- | --- |
| void stan::gm::statement\_visgen::operator() | ( | const no\_op\_statement & |  | ) | const |

inline

Definition at line 1742 of file generator.hpp.

## Member Data Documentation

bool stan::gm::statement\_visgen::include\_sampling\_

Definition at line 1520 of file generator.hpp.

size\_t stan::gm::statement\_visgen::indent\_

Definition at line 1519 of file generator.hpp.

bool stan::gm::statement\_visgen::is\_fun\_return\_

Definition at line 1522 of file generator.hpp.

bool stan::gm::statement\_visgen::is\_var\_

Definition at line 1521 of file generator.hpp.

---

The documentation for this struct was generated from the following file:

- src/stan/gm/generator.hpp


---

[ Stan Home Page ]

*© 2011–2014,
Stan Development Team.*
